# Short-Term Caloric Restriction Attenuates Obesity-Induced Pro-inflammatory Response in Male Rhesus Macaques

**DOI:** 10.3390/nu12020511

**Published:** 2020-02-18

**Authors:** Hollis Wright, Mithila Handu, Allen Jankeel, Ilhem Messaoudi, Oleg Varlamov

**Affiliations:** 1 Omics Data Automation Inc., Beaverton, OR 97005, USA; wrighth@omicsautomation.com; 2 Knight Cancer Institute’s Cancer Early Detection Advanced Research Center, Oregon Health & Science University, Portland, OR 97201, USA; handu@ohsu.edu; 3 Department of Molecular Biology and Biochemistry, School of Biological Sciences, University of California--Irvine, Irvine, CA 92697, USA; allen.jankeel@uci.edu (A.J.); imessaou@uci.edu (I.M.); 4 Division of Cardiometabolic Health, Oregon National Primate Center, Beaverton, OR 97006, USA

**Keywords:** adipose tissue, caloric restriction, high-fat diet, nonhuman primates, obesity, transcriptome, western-style diet

## Abstract

White adipose tissue (WAT) hypertrophy is an essential hallmark of obesity and is associated with the activation of resident immune cells. While the benefits of caloric restriction (CR) on health span are generally accepted, its effects on WAT physiology are not well understood. We previously demonstrated that short-term CR reverses obesity in male rhesus macaques exposed to a high-fat Western-style diet (WSD). Here, we analyzed subcutaneous WAT biopsies collected from this cohort of animals before and after WSD and following CR. This analysis showed that WSD induced adipocyte hypertrophy and inhibited β-adrenergic-simulated lipolysis. CR reversed adipocyte hypertrophy, but WAT remained insensitive to β-adrenergic agonist stimulation. Whole-genome transcriptional analysis revealed that β3-adrenergic receptor and de novo lipogenesis genes were downregulated by WSD and remained downregulated after CR. In contrast, WSD-induced pro-inflammatory gene expression was effectively reversed by CR. Furthermore, peripheral blood monocytes isolated during the CR period exhibited a significant reduction in the production of pro-inflammatory cytokines compared to those obtained after WSD. Collectively, this study demonstrates that short-term CR eliminates an obesity-induced pro-inflammatory response in WAT and peripheral monocytes.

## 1. Introduction

Consumption of a high-fat/calorie-dense Western-style diet (WSD) and physical inactivity are the main risk factors contributing to obesity characterized by a chronic low-grade pro-inflammatory state and insulin resistance [1]. A widely used approach for weight management is caloric restriction (CR). Studies in several organisms, including worms, flies, mice and nonhuman primates (NHPs), have shown that CR promotes survival [2] in part through the extension of health span [3,4]. In humans, CR decreases the risk of cardiovascular and metabolic diseases [5,6,7,8,9] and reduces obesity in older adults [10]. Furthermore, CR has been shown to decrease body weight and adiposity in postmenopausal women [11,12,13]. However, a significant fraction of these women regained weight soon after the termination of dietary intervention [14]. Moreover, we have shown that long-term CR initiated during early adulthood delayed T-cell senescence [15,16]. Specifically, CR initiated during early adulthood preserved circulating naïve CD8 and CD4 T-cells, maintained T-cell receptor diversity, and reduced T-cell production of pro-inflammatory cytokines.

Furthermore, we have previously demonstrated the beneficial effects of CR in obese middle-aged male rhesus macaques. In that study, animals consuming low-fat chow diet (15% calories from fat, 27% from protein, and 59% from carbohydrates) were switched to WSD (33% calories from fat, 17% from protein, 51% from carbohydrates) for six months. Following the WSD period, animals were subjected to a 70% calorically restricted chow diet for an additional four months. These studies showed that CR efficiently reversed WSD-induced obesity and insulin resistance, demonstrating the beneficial effects of short-term CR on metabolic health in NHPs [17,18]. These studies motivated us to further explore transcriptional and functional changes in white adipose tissue (WAT) following WSD-induced weight gain and after CR-induced weight loss. WAT plays a central role in regulating systemic glucose and lipid homeostasis and its dysfunction and the dysregulation of the central control of energy intake contributes to weight regain in obese patients [19]. Thus, we have described the functional and whole-genome RNA sequencing (RNA-Seq) analysis of subcutaneous (SC)-WAT biopsies collected before and after exposure to WSD, and at the end of CR [17,18]. Our study provides new evidence demonstrating that short-term CR is a highly efficient approach for mitigating an obesity-induced pro-inflammatory response and further emphasizes the beneficial effects of CR on immunometabolic health in NHPs.

## 2. Materials and Methods

### 2.1. Animal Characteristics and Diets

**Figure 1:**
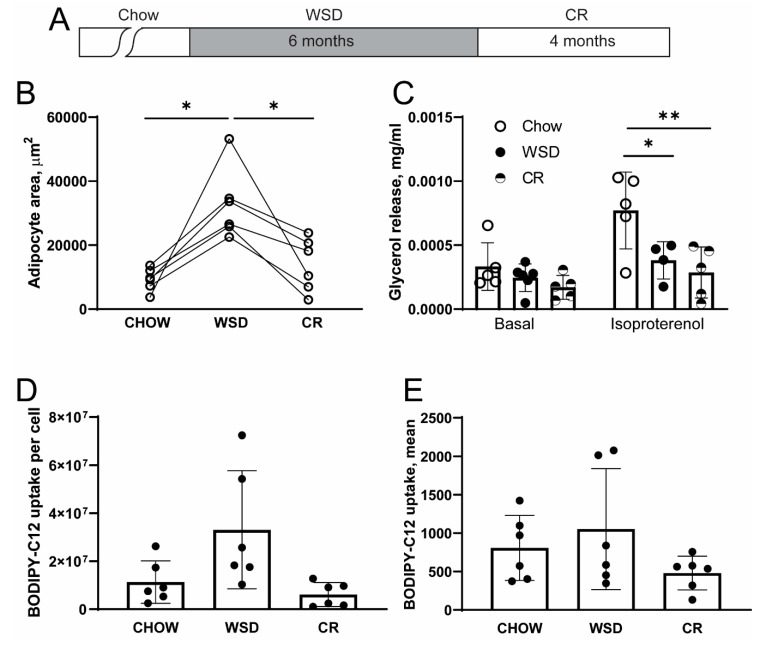
Western-style diet (WSD) induces sustained β-adrenergic resistance in adipocytes. (**A**) Study design. Animals were switched from a chow diet to WSD for six months followed by caloric restriction (CR) for an additional four months. Subcutaneous white adipose tissue (SC-WAT) collected at the end of each dietary intervention (chow, WSD and CR) were separated into small explants and used for lipolysis and free fatty acid (FFA) uptake assays. (**B**) Adipocyte area was determined using BODIPY-C12 labelled explants (see below); each data point is the average cell area per animal for *n* = 80–100 adipocytes; * *p* < 0.05, One-way ANOVA. (**C**) SC-WAT explants were incubated for 2 h under basal conditions, or in the presence of 10 μm isoproterenol and glycerol concentration in the media was determined as described in the “Materials and Methods;” * *p* < 0.05, ** *p* < 0.01, One-way ANOVA. (**D**,**E**) Basal BODIPY-C12 uptake was conducted as described in “Materials and Methods;” (**D**) total fluorescence per adipocyte; (**E**) mean fluorescence per area of adipocyte.

All procedures described in this study were approved by the Oregon National Primate Research Center (ONPRC) Institutional Animal Care and Use Committee. Study design and animal characteristics, including body composition, metabolic status, and cytokine profiles, have been described in our previous study [18]. Briefly, six male rhesus macaques (Indian origin) of 12–13 years of age were housed individually, with the cage size adjusted to animal weight according to the United States Department of Agriculture (USDA) Cage Size Guide, 8th Edition. Individual housing allowed us to mimic a sedentary lifestyle while accurately measuring physical activity and food intake. Chow diet consisted of two daily meals of the Fiber-balanced Monkey Diet (15% calories from fat, 27% from protein, and 59% from carbohydrates; no. 5052; Lab Diet, St. Louis, MO). WSD consisted of two daily meals of the TAD Primate Diet (5LOP) (36% calories from fat, 18% from protein, 45% from carbohydrates, 5A1F, Lab Diet). The Fiber-balanced Monkey Diet contains a lower fraction of high-glycemic carbohydrates and higher levels of low-glycemic carbohydrates compared to the TAD Primate Diet. The study design is outlined in Figure 1A. Before initiation of the study, all animals consumed ad libitum chow diet. Once the animals were individually housed, they were maintained for two months on ad libitum chow diet. During this baseline period, individual caloric intake was determined. After two months on chow diet, animals were switched to ad libitum WSD for six months. The WSD was discontinued after six months when HbA1c values reached prediabetic values [18]. CR was initiated using a chow diet, with the number of chow biscuits adjusted to 70% of individual baseline caloric intake values and continued for four months. CR was stopped after the average SC adipocyte size reached pre-WSD values (Figure 1B). During each dietary intervention, the animals received similar amounts of daily fruit supplements.

### 2.2. WAT Biopsies

Abdominal SC-WAT biopsies were performed by trained surgical personnel at ONPRC according to standard veterinary surgical procedures under sterile conditions and appropriate anesthesia with postoperative pain control, as described [17]. Tissue samples were cut into smaller fragments (explants) with the sharp surgical scissors and used for an ex vivo assay or frozen at −80 °C for RNA isolation.

### 2.3. Cell-Based Assays

Free fatty acid (FFA) uptake, adipocyte size and lipolysis was conducted as previously described [17,20,21,22]. For the lipolysis assay, ~200-mg SC-WAT explants were placed in M199 media (General Electric Company, Boston, MA, USA) at room temperature and transported to the laboratory. 50-mg SC-WAT explants (three basal and three isoproterenol-stimulated replicates) were placed in a 48-well plate containing 0.2 mL incubation medium (Hank’s Salt (HBSS), 0.2% BSA (Sigma-Aldrich, St, Louis, MO, USA) and 5 mM glucose), and incubated at 37 °C for 2 h with or without 10 μM isoproterenol (Sigma-Aldrich, St. Louis, MO, USA). Glycerol release was determined using a glycerol detection kit (Sigma-Aldrich, St. Louis, MO, USA). Glycerol concentrations were normalized to wet tissue weight. For FFA uptake, 100 mg of SC-WAT were collected in M199 media at room temperature and separated into smaller explants. WAT explants were incubated floating in a 48-well plate filled with 0.4 mL incubation medium (M199 medium, 0.1% fatty acid-free BSA (Sigma-Aldrich, St. Louis, MO, USA), 20 mM HEPES (pH 7.4)). Fluorescently-labeled fatty acid BODIPY-500/510 C_1_, C_12_ (BODIPY-C12; Life Technologies, Waltham, MA, USA) was prepared by diluting a 2.5 mM methanol stock solution in incubation medium to a final concentration of 10 μM and incubated for 15 min in a 37 °C water bath. One hundred μL of diluted BODIPY-C12 and 2 μL of live-cell staining dye Calcein Red-Orange AM (Life Technologies, Waltham, MA, USA) were added to each well and WAT explants were incubated for 15 min at 37 °C. Media was removed and explants were washed three times with incubation medium. WAT explants were fixed at room temperature in 4% paraformaldehyde (Sigma-Aldrich, St. Louis, MO, USA)/PBS for 20 min, washed with PBS and analyzed by confocal microscopy. WAT explants were placed into a 35-mm glass-bottom imaging culture dish (MatTek, Ashland, MA, USA) in PBS. Confocal microscopy was performed using a Leica SP5 AOBS spectral confocal system supplied with a ×20 objective. Adipocyte area and BODIPY fluorescence were measured using Fiji imaging software. The statistical analysis of adipocyte parameters was conducted using repeated measure one-way ANOVA followed by the Tukey’s multiple comparison test using GraphPad Prism 8.2.0.

### 2.4. RNA-Seq Analysis

200 mg of frozen SC-WAT samples were homogenized with a TissueLyzer-II (QIAGEN, Hilden, Germany). RNA was isolated using the AllPrep DNA/RNA purification kit (QIAGEN). High-quality RNA (RIN >8) was used for library construction. RNA-Seq libraries were prepared using the TruSeq protocol (Illumina, San Diego, CA, USA) as described in [18]. Poly (A) + RNA was purified using oligo-dT coated magnetic beads and chemically fragmented followed by cDNA generation using random hexamer primers. The cDNA ends were repaired and ligated to library adaptors. Following purification with AMPure XP beads (Beckman Coulter Inc., Brea, CA, USA), libraries were amplified using 11 PCR cycles. Amplified libraries were cleaned using AMPure XP beads. Libraries were analyzed on a Bioanalyzer (Agilent, Santa Clara, CA, USA) and quantified using qRT-PCR (Kapa Biosystems, Wilmington, MA, USA) on a StepOnePlus qRT-PCR workstation (Life Technologies, Carlsbad, CA, USA). Libraries were multiplexed and final concentrations were determined by qRT-PCR. Multiplexed libraries were diluted to 1 nM for denaturation and then diluted to deliver optimal clustering on the flow cell. Flow cells were prepared on a cBot (Illumina, San Diego, CA, USA). Libraries were sequenced on a HiSeq 2000 (Illumina, San Diego, CA, USA). Data was assembled into standard fastq files using Bcl2Fastq (Illumina, San Diego, CA, USA). The quality of the raw reads was verified using FastQC (version 0.11.3). Low quality bases as well as any remaining Illumina adapters were trimmed. Reads with less than 25 bases remaining were discarded. The remaining reads were aligned to the rhesus macaque genome (genome assembly: Macaca mulatta 1.0) from ENSEMBL using the splice aware short read aligner suite Bowtie2/TopHat2 [23] in a strand-specific fashion allowing up to 5% mismatches. Reads were assigned to features using the featureCounts function of the Rsubread package [24] in R 3.4, using default parameters except for setting allowMultiOverlaps to TRUE. Transcripts were normalized and analyzed using the glm functionality of edgeR [25]; the candidate differentially expressed genes (DEGs) were identified as those genes with a fold change (FC) ≥ 2 and a false discovery rate (FDR) ≤ 0.05.

### 2.5. Bioinformatic Analysis

Differential gene expression results (fold change, *p*-value, and FDR) were imported into the Ingenuity Pathway Analysis program (IPA, QIAGEN, Hilden, Germany). To facilitate this, ENSEMBL rhesus gene IDs were first mapped to human gene IDs using Ensembl’s BioMart. Only high-confidence matches were used, and only the top match was chosen when one rhesus ID mapped to many human gene IDs. If no human gene ID was found, the rhesus gene ID was retained. Once imported into IPA, a fold-change cutoff of 1.5 and an FDR cutoff of 0.05 was applied to all comparisons to select differentially expressed genes. The STRING protein-protein interaction analysis (https://string-db.org/) was performed by pasting ENSEMBL IDs of DEGs into the STRING input window. The evidence-based STRING analysis was performed using the medium confidence value of 0.4.

### 2.6. Flow Cytometry Analysis

To measure frequencies of lymphocyte subsets, peripheral blood mononuclear cells (PBMCs) were surface-stained with antibodies against CD8β (Beckman Coulter, Brea, CA, USA), CD4 (eBioscience, San Diego, CA, USA), CD28 (BioLegend, San Diego, CA, USA), CD95 (BioLegend, San Diego, CA, USA), CCR7 (BD Pharmingen, San Diego, CA, USA), CD20 (BioLegend, San Diego, CA, USA), IgD (Southern Biotech, Birmingham, AL, USA), and CD27 (BioLegend, San Diego, CA, USA). This combination allowed for the delineation of central memory (CM; CD28 + CD95 + CCR7 +), transitional effector memory (TEM; CD28 + CD95 + CCR7 −), and effector memory (EM; CD28− CD95+ CCR7−), CD4 and CD8 T-cells (as well as marginal zone-like (MZ-like; IgD + CD27 +), memory (IgD − CD27 +), and double-negative (DN; IgD − CD27 −) B cell subsets). A second tube of PBMCs was stained with antibodies against CD3 (BD Pharmingen, San Diego, CA, USA), CD20 (Beckman Coulter, Brea, CA, USA), CD14 (BioLegend, San Diego, CA, USA), HLA-DR (BioLegend, San Diego, CA, USA), CD11c (BioLegend, San Diego, CA, USA), CD123 (BioLegend, San Diego, CA, USA), and CD8α (BD Pharmingen, San Diego, CA, USA) to delineate monocytes (CD3 − CD20 − CD14 + HLA-DR +), dendritic cells (DCs; CD3 − CD20 − CD14 − HLA-DR +), and natural killer (NK) cells (CD3 − CD20 − CD8a +). DCs were further defined into myeloid (mDCs; CD123 − CD11c +) and plasmacytoid (pDCs; CD123 + CD11c −) DCs. To measure inflammatory cytokine production by monocytes, PBMCs were stimulated with lipopolysaccharide (LPS) (100 μg/mL, PeproTech, Rocky Hill, NJ, USA) in the presence of brefeldin A (Sigma, St. Louis, MO, USA) for 6 h. After incubation, cells were stained with antibodies directed against CD4, CD8β, CD14, HLA-DR, and CD20. Samples were then fixed, permeabilized (permeabilization buffer, BioLegend, San Diego, CA, USA), and stained using antibodies against interferon γ (IFNγ, eBioscience, San Diego, CA, USA), tumor necrosis factor α (TNFα, eBioscience, San Diego, CA, USA), and interleukin-6 (IL-6, BioLegend, San Diego, CA, USA). All samples were analyzed using the Attune NxT Flow Cytometer (LIfeTech, Carlsbad, CA, USA) and FlowJo software (TreeStar, Ashland, OR, USA). The statistical analysis of flow cytometry data was conducted using the paired *t*-test.

## 3. Results

### 3.1. WSD-Induced Adipocyte Hypertrophy but not β-Adrenergic Resistance is Reversed by CR

In our previous studies, we demonstrated that adult male rhesus macaques exposed to six months of WSD exhibited a significant increase in fat mass. Furthermore, fat mass was decreased significantly following the subsequent CR period. We also determined that the consumption of a WSD induced systemic insulin resistance, while CR restored normal insulin sensitivity in rhesus macaques [17,18]. The analysis of SC-WAT biopsies collected during each dietary period showed that WSD led to a significant increase in the mean area of SC adipocytes, while subsequent CR reduced adipocyte area to the baseline levels (Figure 1B). To assess the effect of diet on lipolysis, SC-WAT explants derived from three longitudinal biopsies were incubated ex vivo in basal media or in the presence of the β-adrenergic receptor agonist isoproterenol, and glycerol release in incubation media was quantified. SC-WAT collected during the WSD period exhibited a blunted β-adrenergic response compared to a chow diet, while the basal lipolysis remained unchanged. Interestingly, CR failed to restore a normal β-adrenergic response in SC-WAT despite a significant reduction in adipocyte area observed at the end of the CR period (Figure 1C). To assess the uptake of FFA in adipocytes, SC-WAT explants were incubated with the metabolizable fluorescent FFA analogue BODIPY-C12 [21,22,26,27,28] and single-cell fluorescence was quantified by confocal microscopy. SC adipocytes from WSD biopsies exhibited a trend to higher FFA uptake compared to the chow control and the CR conditions (Figure 1D,E). Collectively, this study shows that WSD induces SC adipocyte hypertrophy, which was efficiently reduced to a pre-obese level by CR. In contrast, WSD led to the inhibition of the β-adrenergic lipolytic response in SC adipocytes and this effect persisted even after adipocyte size, body weight and insulin sensitivity [17,18] were normalized by CR.

### 3.2. WSD-Induced Pro-Inflammatory Gene Expression in SC-WAT Is Reversed by CR

**Figure 2:**
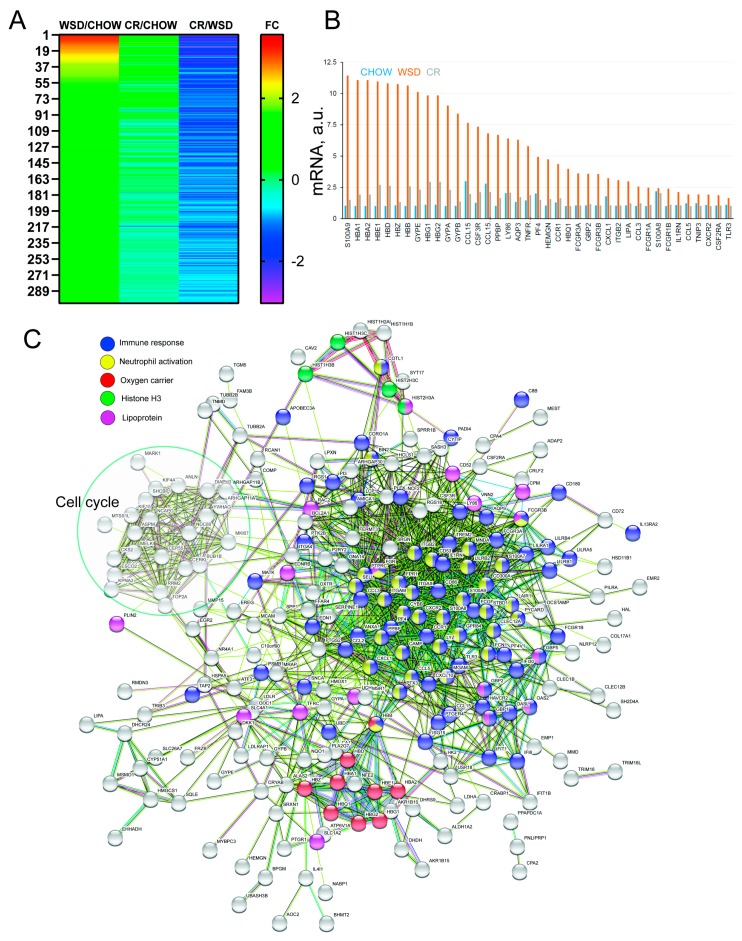
Western-style diet (WSD) induces and caloric restriction (CR) reverses pro-inflammatory gene expression in subcutaneous white adipose tissue (SC-WAT). Three SC-WAT biopsies collected at the end of each dietary period were subjected to the RNA-Seq transcriptional analysis. This figure describes only reversibly-induced differentially expressed genes (DEGs) that were significantly upregulated by WSD and then significantly downregulated by CR. (**A**) Heat map of DEGs (the number of genes is indicated); FC, log2fold changes in gene expression. (**B**) Top WSD-induced DEGs that were downregulated by CR; relative mRNA levels are shown in arbitrary units (a.u.). (**C**) STRING Functional Protein Network Analysis of DEGs reveals functional connectivity associated with immune response (blue), neutrophil activation (yellow), heme biosynthesis (red), histone H3 network (green), lipoprotein metabolism (magenta), and regulation of cell cycle progression (circled).

Three SC-WAT biopsies collected at the end of each dietary period were subjected to whole-genome RNA-Seq transcriptional analysis. DEGs were identified using three comparisons: WSD/CHOW, CR/WSD, and CR/CHOW (Supplementary Table S1). This experimental design allowed us to identify diet-specific DEGs, as well as genes whose expression did not change after the transition from WSD to CR. Functional enrichment was performed on WSD/CHOW, CR/WSD, and CR/CHOW subsets using IPA. Additionally, we identified 301 DEGs whose expression was significantly upregulated by WSD, then subsequently significantly downregulated by CR (Figure 2A and Supplementary Table S2). DEGs of this category with the highest fold change encoded pro-inflammatory factors such as *S100A7/8*, *CXCL1*, *CCL3*, *CCL5* and *MMD*; chemokine receptors e.g., *CCR1* and *CXCR2*, as well as proteins involved in the heme biosynthesis pathway, such as *ALAS2*, *HBB*, *HBA1* and *CYPE* (Table 1). WSD induced the upregulation of genes involved in granulocyte (*p*-value 4.2 × 10^−12^) and agranulocyte (*p*-value 8.1 × 10^−10^) migration and cell adhesion. The top upstream regulators identified by IPA included the pro-inflammatory cytokines *TNF*α (*p*-value 4.5 × 10^−21^) and interleukin-1β (*IL-1*β, *p*-value 4.8 × 10^−19^). To identify the functional interaction networks between proteins encoded by DEGs, we used the STRING Functional Protein Network Analysis. This analysis revealed the largest node of functional connectivity associated with immune response (*n* = 85; FDR 3.7 × 10^−23^) and neutrophil activation (*n* = 34; FDR 3.3 × 10^−11^) (Figure 2C, blue and yellow). Furthermore, we identified three functional nodes representing oxygen transport processes (*n* = 9; FDR 1.3 × 10^−8^), the histone H3 network (*n* = 4, FDR 0.01), and lipoprotein metabolism (*n* = 23; FDR 0.02) (Figure 2C and Table 2, “LDLR”). Intriguingly, STRING identified a well-segregated node of functionally interacting proteins implicated in the regulation of cell cycle progression, DNA replication and intracellular signaling (Figure 2C, “Cell Cycle”). Collectively, this analysis suggests that WSD induces a pro-inflammatory transcriptional response in SC-WAT that is effectively reversed by subsequent CR.

### 3.3. De Novo Lipogenesis and β-Adrenergic Receptor Genes Remain Downregulated after CR

**Figure 3:**
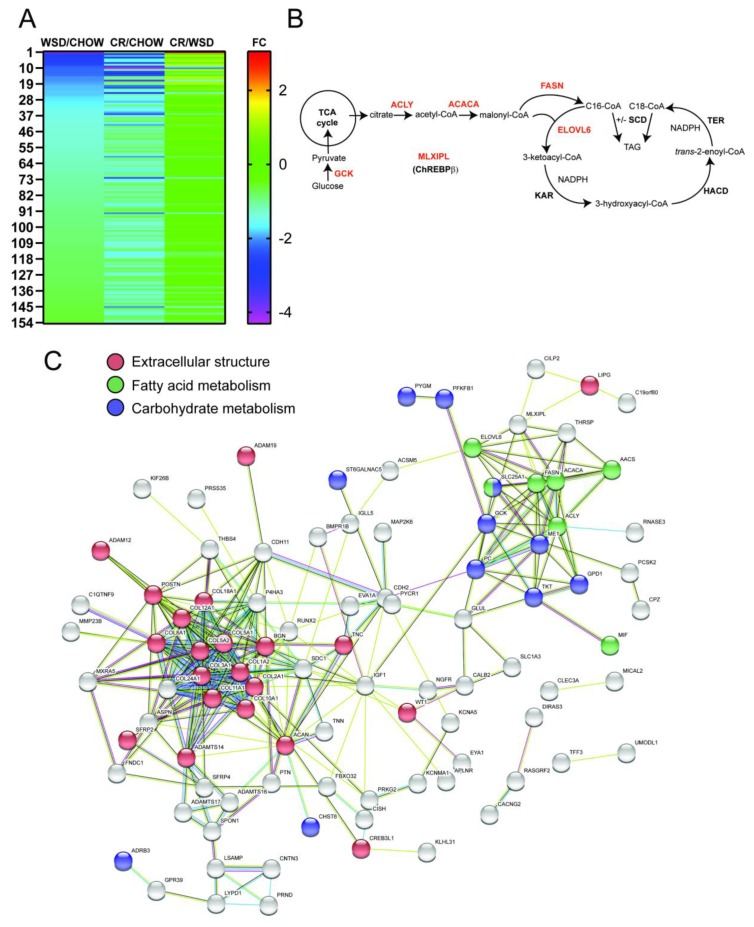
Western-style diet (WSD) causes sustained downregulation of de novo lipogenesis (DNL) genes in subcutaneous white adipose tissue (SC-WAT). Three SC-WAT biopsies collected at the end of each dietary period were subjected to the RNA-Seq transcriptional analysis. This figure describes a subset of differentially expressed genes (DEGs) that were downregulated after WSD and remained downregulated following caloric restriction (CR). (**A**) Heat map of DEGs (the number of genes of this category is indicated); FC, log2fold changes in gene expression. (**B**) DNL pathway; enzymes and transcription factors are in red: glucokinase (GCK), ATP-citrate lyase (ACLY), acetyl-CoA carboxylase (ACACA), fatty acid synthase (FASN), carbohydrate-responsive-element-binding protein-beta (MLXIPL/ChREBPβ, the transcriptional regulator of DNL). DNL-derived palmitate (C16) is elongated (C16− > C18) by elongation of very long chain fatty acids protein 6 (ELOVL6). C16 and C18 are desaturated by stearoyl-CoA desaturases (SCD). Additional enzymes regulating fatty acid elongation: 3-ketoacyl-CoA reductase (KAR), trans-2-enoyl-CoA reductase (TER), 3-hydroxyacyl ACP dehydrase (HACD); TAG, triacylglycerol. (**C**) STRING Functional Protein Network Analysis of downregulated DEGs reveals functional connectivity associated with fatty acid metabolism/DNL (green), carbohydrate metabolism (blue), and extracellular matrix structure (red).

Gene expression analysis revealed a subset of 153 DEGs whose expression was significantly downregulated by WSD (FC < −1.5; FDR < 0.05) and remained significantly downregulated even after the CR period (FDR < 0.05; Figure 3A and Supplementary Table S3). The IPA pathway analysis showed that WSD caused the downregulation of genes encoding enzymes involved in de novo FFA biosynthesis from glucose (de novo lipogenesis, DNL), including ATP citrate lyase (*ACLY*)*,* acetyl-coenzyme A carboxylase alpha (*ACACA*)*,* FFA synthase (*FASN*)*,* and elongation of very long chain FFAs protein 6 (*ELOVL6*) (Figure 3B and Table 2). In addition, carbohydrate metabolism genes of glucokinase (*GCK*), 6-phosphofructo-2-kinase/fructose-2, 6-bisphosphatase 1 (*PFKFB1*) and glycogen phosphorylase (*PYGM*) were also downregulated after WSD and remained downregulated even after CR (Figure 3B and Table 2). Changes in the expression of DNL genes correlated with the sustained downregulation of the gene encoding the master regulator of DNL, carbohydrate-responsive-element-binding protein (*ChREBP, MLXIPL*) [29,30] observed both after the WSD and following CR (WSD/Chow, 1.8 fold change, FDR 0.00067; Figure 3B,C). Furthermore, genes mediating the lipolytic function in adipocytes, including β*_3_-*adrenergic receptor (*ADRB3*) [31] and G Protein-Coupled Receptor 39 (*GPR39*) [32,33] were significantly downregulated after WSD compared to chow (FDR 8.6 × 10^−5^) and remained downregulated even after CR (Figure 3C and Table 2). The STRING Functional Protein Network Analysis revealed the downregulation of functional nodes related to FFA (*n* = 7; FDR 0.0033) and carbohydrate (*n* = 13; FDR 0.0012) metabolism and collagen biosynthesis (*n* = 22; FDR 1.0 × 10^−11^) (Figure 3C). Collectively, our analysis shows that WSD induced the downregulation of DNL and lipolysis genes, which persisted even after CR-induced reduction in SC adipocyte size.

### 3.4. CR Diminishes a Pro-Inflammatory Response in Circulating Monocytes

**Figure 4:**
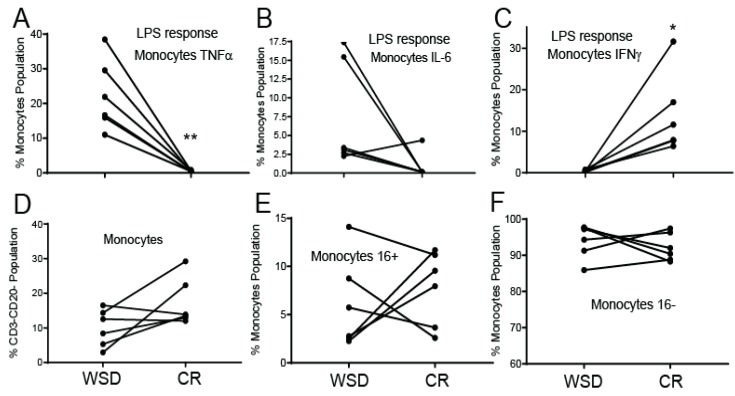
Caloric restriction (CR) suppresses a pro-inflammatory response in circulating monocytes. (**A**–**C**) Frequency of TNFα, IL-6, and IFNγ producing monocytes following 6 h liposaccharide (LPS) stimulation as measured by intracellular cytokine staining. (**D**) Frequency of monocytes within PBMCs. (**E**,**F**) Relative frequency of non-classical (CD16+) and classical (CD16-) monocytes within the monocyte population; * *p* < 0.05, ** *p* < 0.01, paired *t*-test.

Obesity is associated with the development of a systemic pro-inflammatory state and the production of pro-inflammatory cytokines. In contrast, CR typically exerts the opposite effect on the immune system, leading to a reduced pro-inflammatory response [34], although the later has not been directly demonstrated in a NHP model of CR. To examine these dietary effects further, PBMCs isolated after exposure to WSD and at the end of CR were treated with the toll-like receptor-4 (TLR4) agonist LPS and the frequency of TNFα- producing monocytes was quantified using intracellular staining and flow cytometry. Approximately 10–40% of the total monocyte population isolated during the WSD period were TNFα-positive following LPS stimulation. In contrast, very few monocytes isolated at the end of the CR period expressed TNFα following LPS stimulation, suggesting that fasting supported the loss of obesity-induced pro-inflammatory phenotype in circulating monocytes (Figure 4A). Similar results were obtained using another pro-inflammatory cytokine IL-6, which was also downregulated in monocytes by CR (Figure 4B). In contrast to TNFα and IL-6, the percent of IFNγ + monocytes dramatically increased following a transition from WSD to CR (Figure 4C), suggesting that WSD suppressed and CR supported the production of a cytokine that is critical for antiviral and antibacterial immunity. Interestingly, a transition from WSD to CR did not cause significant changes in the proportion of total circulating monocytes or the ratio of classical (CD16−) to non-classical (CD16+) monocyte subtypes in peripheral blood (Figure 4D–F), suggesting that CR may modulate the functional capacity of the cells rather than cause major shifts in monocyte population frequencies.

## 4. Discussion

### 4.1. The Effects of WSD and CR on a Pro-Inflammatory Response

The main finding of the present report is that short-term CR efficiently reversed an obesity-induced pro-inflammatory transcriptional response in SC-WAT and reduced the production of pro-inflammatory cytokines in LPS-treated peripheral monocytes. The latter finding is consistent with the reported reduction in the cytokine levels in LPS-treated T-cell populations isolated from long-term calorically restricted rhesus macaques [16,34], suggesting that fasting can have a beneficial effect on both adaptive and innate immune systems. It is important to emphasize that previous NHP studies were conducted using standard monkey chow low in saturated fats [16,34], while we used WSD matching the composition of a typical diet consumed in a modern society. Since WAT inflammation is strongly associated with insulin resistance [35], the present study suggests that a relatively short-term energy restriction can efficiently mitigate obesity-induced insulin resistance and reverse a pro-inflammatory phenotype in both SC-WAT and peripheral monocytes. Studies shows that the key pro-inflammatory cytokines driving insulin resistance are TNFα and IL1β secreted by local macrophages [36]. Furthermore, the suppression of a pro-inflammatory response by CR has been attributed to the production of ketone bodies and acetoacetate in the liver as alternative energy sources during nutrient deficiency [37]. The anti-inflammatory effect of ketogenic bodies has been linked to the direct inhibition of the NLRP3 inflammasome in human monocytes stimulated with LPS [38], which is consistent with the present and previous reports [16,34] that CR causes a significant reduction in the production of pro-inflammatory cytokines in rhesus monocytes and T-cells, respectively. The induction of erythroid-specific genes in SC-WAT by WSD reported here is a new phenomenon. Although the expression of *HBB* in mouse macrophages has been previously reported [39], the present study shows that WSD induces and CR reverses the expression of multiple erythroid-specific genes, including embryonic hemoglobin genes *HBZ* and *HBE1*. To our knowledge, this is the first report that shows a diet-specific regulation of erythroid genes in peripheral WAT. This may represent an increased production and/or bone marrow output of the erythroid progenitors in the presence of WSD. This hypothesis is consistent with our previous study demonstrating that WSD induced the expression of erythroid genes in the skeletal muscle of rhesus macaques [18].

### 4.2. The Effects of WSD and CR on Lipolysis

The present study demonstrates that male rhesus macaques transitioned from a low-fat diet to a WSD for six months exhibited SC adipocyte hypertrophy, a blunted β-adrenergic lipolytic response, and the downregulation of the *ADRB3* gene in SC-WAT. While CR efficiently reduced SC adipocyte size, it did not normalize the β-agonist lipolytic response and failed to restore *ADRB3* expression in SC-WAT. Several studies have shown that obese patients undergoing weight loss interventions experience weight regain [40,41,42], which coincides with functional changes in WAT [43]. Consistent with the present report, obesity has been shown to be associated with the development of lipolytic resistance of WAT to catecholamines [44]. A number of studies, however, have also demonstrated that CR increases the rate of catecholamine response and the density of β-adrenoceptors in WAT of obese patients [45,46,47], while others reported no significant effects of CR on lipolysis [48,49,50,51,52]. Interestingly, studies have shown that, during the weight-maintenance phase after the bariatric surgery, there was a marked decrease in the levels of hormone-sensitive lipase (HSL) in SC-WAT of women but not in men [53]. Similarly to the present study, it has been shown that the WAT lipolytic response of obese patients three years after initiation of a weight-reducing program was lower compared to the pre-diet conditions [54]. These studies demonstrate that the initial improvement of the lipolytic response after weight loss persists during the dynamic phase of energy restriction, but returns to lower values after adaptation to a new energy balance during the weight-maintenance phase. We also observed the sustained downregulation of *GPR39* in SC-WAT by WSD. *GPR39* deficiency has been reportedly associated with obesity and reduced lipolysis in adipocytes [32,33].

### 4.3. The Effects of WSD and CR on De Novo Lipogenesis Pathway

The present study demonstrates that DNL and *ChREBP* genes, whose expression in SC-WAT was suppressed by WSD, remained downregulated after CR. Previously, it has been shown that the WAT-specific isoform of the transcription factor ChREBP, ChREBPβ, is the central regulator of glucose-mediated activation of lipogenic genes, while its expression in WAT correlates with increased insulin sensitivity [29]. ChREBP activation in WAT is altered in obesity, resulting in the downregulation of *ChREBP*β, *ACLY, ACACA, FASN,* and *ELOVL6* [55]. Activation of DNL in WAT has a positive effect both on direct glucose disposal [56,57] and metabolic homeostasis through the production of lipokines [58,59]. While the adipose-specific DNL pathway significantly contributes to body lipid reserves and thus directly regulates glucose homeostasis in humans [58], new studies have shown that it is also involved in the synthesis of biologically active branched FFAs that participate in the regulation of systemic insulin sensitivity and have anti-inflammatory properties [60,61]. The metabolic role of DNL-derived lipokines, in particular palmitoleate (C16: 1n−7), has been extensively described in a recent review [59]. The lipid biosynthesis pathway is dysregulated in obesity, which has been associated with the remodeling of lipid composition of adipocyte membranes [62] and the reduced presence of polyunsaturated fatty acids in high-BMI individuals [63]. Thus, we propose that WSD exerts a long-lasting effect on the lipolytic and DNL pathways in adipocytes, possibly through epigenetic modifications. Consistent with this hypothesis, we recently conducted the whole-genome gene expression and epigenetic analysis in visceral WAT of female rhesus macaques exposed to a WSD and excess androgens in combination or individually. This study demonstrated that testosterone could modulate the effects of WSD on transcription of lipid metabolism genes and DNA methylation in females WAT [64].

## 5. Conclusions

In summary, the present study advances our understanding of the functional and transcriptional response in SC-WAT associated with dynamic changes in fat mass in response to diet-induced obesity and weight loss and expands our previous studies in NHPs [17,18,20,64,65,66]. Our study has several limitations. In the present report, we used the RNA-Seq analysis of whole WAT. Further studies are needed to understand the immunophenotypic changes in WAT (in both SC and visceral depots) in the content of resident immune cells. While several previous reports addressed the metabolic adaptation of WAT to weight loss through energy restriction [67,68,69], sex-specific mechanisms that control WAT function in health, during the development of obesity and after weight loss are also not completely understood [31,70] and need to be further explored in a NHP model. Further studies are needed to understand the role of epigenetic factors, including DNA methylation and histone modifications, in programing WAT transcriptome in males and females.

## Figures and Tables

**Table 1:** Top western-style diet (WSD)-induced genes whose expression was reversed by caloric restriction (CR).

		WSD/CHOW		CR/CHOW		CR/WSD	
Ensembl_ID	Entrez Gene Name	LogFC	FDR	LogFC	FDR	LogFC	FDR
ALAS2	5’-aminolevulinate synthase 2	3.56	3 × 10^−52^	1.24	2 × 10^−4^	−2.33	1 × 10^−19^
HBZ	hemoglobin subunit zeta	3.35	7 × 10^−49^	0.79	0.066	−2.56	5 × 10^−23^
HBD	hypophosphatemic bone disease	3.14	2 × 10^−47^	1.28	4 × 10^−5^	−1.86	5.3 × 10^−13^
HBB	hemoglobin subunit beta	3.11	1 × 10^−46^	1.26	5 × 10^−5^	−1.85	5.7 × 10^−13^
HBA1	hemoglobin subunit alpha 1	3.09	1 × 10^−46^	0.87	0.013	−2.21	1.7 × 10^−18^
HBA2	hemoglobin subunit alpha 2	3.09	1 × 10^−46^	0.87	0.013	−2.21	1.8 × 10^−18^
HBE1	hemoglobin subunit epsilon 1	3.08	1.7 × 10^−45^	1.23	9 × 10^−5^	−1.85	5.6 × 10^−13^
S100A9	S100 calcium binding protein A9	3.06	4 × 10^−35^	0.54	0.516	−2.53	1.4 × 10^−18^
PF4	platelet factor 4	2.98	4.8 × 10^−22^	1.70	1 × 10^−4^	−1.28	0.00054
GYPE	glycophorin E	2.87	4 × 10^−32^	1.00	0.02	−1.87	2 × 10^−10^
GYPA	glycophorin A	2.80	5.7 × 10^−35^	1.11	0.002	−1.69	1.4 × 10^−9^
HBBP1	hemoglobin subunit beta pseudogene 1	2.75	3.3 × 10^−7^	−0.28	1	−3.03	1.1 × 10^−6^
HBG1	hemoglobin subunit gamma 1	2.63	3.3 × 10^−8^	1.05	0.021	−1.58	2.8 × 10^−7^
HBG2	hemoglobin subunit gamma 2	2.63	3.6 × 10^−23^	1.05	0.021	−1.58	2.8 × 10^−7^
GYPB	glycophorin B (MNS blood group)	2.58	1 × 10^−19^	0.46	0.84	−2.12	6.3 × 10^−10^
CXCL1	C-X-C motif chemokine ligand 1	2.38	1.2 × 10^−13^	1.13	0.095	−1.25	0.00614
S100A8	S100 calcium binding protein A8	2.28	1 × 10^−20^	0.07	1	−2.21	3.7 × 10^−13^
S100A7	S100 calcium binding protein A7	1.69	0.028	−0.03	1	−1.72	0.0441
MSR1	macrophage scavenger receptor 1	1.50	7.5 × 10^−13^	0.51	0.231	−1.00	0.000509
CCL5	C-C motif chemokine ligand 5	1.44	3.3 × 10^−7^	0.40	0.819	−1.03	0.010258
CCR1	C-C motif chemokine receptor 1	1.30	7 × 10^−9^	0.25	0.901	−1.04	0.000282
CXCR2	C-X-C motif chemokine receptor 2	1.25	7.2 × 10^−6^	0.23	0.995	−1.02	0.006017
CXCL10	C-X-C motif chemokine ligand 10	1.24	1.5 × 10^−5^	0.10	1	−1.14	0.004538
CCL3	C-C motif chemokine ligand 3	1.14	4 × 10^−6^	0.38	0.712	−0.77	0.040866
MMD	monocyte to macrophage differentiation associated	1.13	6.7 × 10^−8^	−0.27	0.86	−1.41	4.3 × 10^−8^

Differentially expressed genes (DEGs) were filtered using the following parameters. WSD/CHOW, Log_2_ (fold change) > 1.0 and false discovery rate (FDR) < 0.05; CR/WSD, FDR < 0.05.

**Table 2:** Top metabolic genes differentially regulated by western-style diet (WSD) and caloric restriction (CR).

		WSD/CHOW		CR/CHOW		CR/WSD	
Ensembl_ID	Entrez Gene Name	LogFC	FDR	LogFC	FDR	LogFC	FDR
ELOVL6	ELOVL fatty acid elongase 6	−2.203	0.000	−2.959	0.000	−0.756	0.023
FASN	fatty acid synthase	−1.880	0.000	−2.139	0.000	−0.259	0.720
ACLY	ATP citrate lyase	−1.469	0.000	−2.310	0.000	−0.841	0.003
ACACA	acetyl-CoA carboxylase alpha	−1.384	0.000	−1.936	0.000	−0.552	0.133
ADCY10	adenylate cyclase 10 (soluble)	−1.251	0.000	−1.970	0.000	−0.719	0.044
GPR39	G protein-coupled receptor 39	−1.200	0.000	−1.208	0.000	−0.008	1.000
RUNX2	runt related transcription factor 2	−1.100	0.000	−0.771	0.021	0.329	0.621
PLIN2	perilipin 2	1.546	0.000	0.350	0.623	−1.196	0.000
LDLR	low density lipoprotein receptor	1.555	0.000	−0.491	0.284	−2.046	0.000

Differentially expressed genes (DEGs) were filtered using the following parameters. WSD/CHOW, Log_2_ (fold change) < 1.0 and FDR < 0.05; CR/CHOW, Log_2_ (fold change) < 1.0 and FDR < 0.05. PLIN2 and LDLR show the reversible pattern of regulation (upregulated by WSD and downregulated by CR).

## Supplementary Materials

The following are available online at https://www.mdpi.com/2072-6643/12/2/511/s1, Table S1: Title: Diet-specific changes in gene expression, Table S2: Title: Top 200 WSD-induced genes whose expression was reversed by CR, and Table S3: Title: WSD-suppressed genes whose expression was not reversed by CR.

## Online Data Repository

Sequencing data are available at the Sequence Read Archive (accession no. PRJNA606903).

## References

[1] Kahn S.E., Hull R., Utzschneider K.M. (2006). Mechanisms linking obesity to insulin resistance and type 2 diabetes. Nature.

[2] Varlamov O. (2017). Western-style diet, sex steroids and metabolism. Biochim. et Biophys. Acta (BBA)-Mol. Basis Dis..

[3] Mirzaei H., Suarez J.A., Longo V.D. (2014). Protein and amino acid restriction, aging and disease: From yeast to humans. Trends Endocrinol. Metab..

[4] Lee C., Longo V.D. (2016). Dietary restriction with and without caloric restriction for healthy aging. F1000Research.

[5] Heilbronn L.K., de Jonge L., Frisard M.I., DeLany J.P., Larson-Meyer D.E., Rood J., Nguyen T., Martin C.K., Volaufova J., Most M.M. (2006). Effect of 6-month calorie restriction on biomarkers of longevity, metabolic adaptation, and oxidative stress in overweight individuals: A randomized controlled trial. JAMA.

[6] Larson-Meyer D.E., Heilbronn L.K., Redman L.M., Newcomer B.R., Frisard M.I., Anton S., Smith S.R., Maplstat A.A., Ravussin E. (2006). Effect of Calorie Restriction With or Without Exercise on Insulin Sensitivity, β-Cell Function, Fat Cell Size, and Ectopic Lipid in Overweight Subjects. Diabetes Care.

[7] Redman L.M., Heilbronn L.K., Martin C.K., Alfonso A., Smith S.R., Ravussin E. (2007). Effect of Calorie Restriction with or without Exercise on Body Composition and Fat Distribution. J. Clin. Endocrinol. Metab..

[8] Fontana L., Villareal D.T., Weiss E.P., Racette S., Steger-May K., Klein S., Holloszy J.O. (2007). Calorie restriction or exercise: Effects on coronary heart disease risk factors. A randomized, controlled trial. Am. J. Physiol. Metab..

[9] Fontana L. (2009). The scientific basis of caloric restriction leading to longer life. Curr. Opin. Gastroenterol..

[10] Villareal D.T., Chode S., Parimi N., Sinacore D.R., Hilton T., Armamento-Villareal R., Napoli N., Qualls C., Shah K. (2011). Weight loss, exercise, or both and physical function in obese older adults. New Engl. J. Med..

[11] Katsoulis K., Blaudeau T.E., Roy J.P., Hunter G.R. (2009). Diet-induced Changes in Intra-abdominal Adipose Tissue and CVD Risk in American Women. Obesity (Silver Spring).

[12] Camhi S.M., Stefanick M.L., Katzmarzyk P.T., Young D.R. (2010). Metabolic syndrome and changes in body fat from a low-fat diet and/or exercise randomized controlled trial. Obesity (Silver Spring).

[13] Foster-Schubert K.E., Alfano C.M., Duggan C.R., Xiao L., Campbell K.L., Kong A., Bain C.E., Wang C.Y., Blackburn G.L., McTiernan A. (2012). Effect of diet and exercise, alone or combined, on weight and body composition in overweight-to-obese postmenopausal women. Obesity (Silver Spring).

[14] Carels R.A., Darby L.A., Cacciapaglia H.M., Douglass O.M. (2004). Reducing Cardiovascular Risk Factors in Postmenopausal Women through a Lifestyle Change Intervention. J. Women’s Heal..

[15] Messaoudi I., Warner J., Fischer M., Park B., Hill B., Mattison J., Lane M.A., Roth G.S., Ingram N.K., Picker L.J. (2006). Delay of T cell senescence by caloric restriction in aged long-lived nonhuman primates. Proc. Natl. Acad. Sci. USA.

[16] Messaoudi I., Fischer M., Warner J., Park B., Mattison J., Ingram N.K., Totonchy T., Mori M., Nikolich-Zugich J. (2008). Optimal window of caloric restriction onset limits its beneficial impact on T-cell senescence in primates. Aging Cell.

[17] Cameron J.L., Jain R., Rais M., White A.E., Beer T.M., Kievit P., Winters-Stone K., Messaoudi I., Varlamov O. (2016). Perpetuating effects of androgen deficiency on insulin resistance. Int. J. Obes..

[18] Messaoudi I., Handu M., Rais M., Sureshchandra S., Park B.S., Fei S., Wright H., White A.E., Jain R., Cameron J. (2017). Long-lasting effect of obesity on skeletal muscle transcriptome. BMC Genom..

[19] MacLean P., Higgins J.A., Giles E.D., Sherk V.D., Jackman M.R. (2015). The role for adipose tissue in weight regain after weight loss. Obes. Rev..

[20] Varlamov O., Bishop C., Handu M., Takahashi D., Srinivasan S., White A., Roberts C.T. (2017). Combined androgen excess and Western-style diet accelerates adipose tissue dysfunction in young adult, female nonhuman primates. Hum. Reprod..

[21] Varlamov O., Chu M., Cornea A., Sampath H., Roberts C.T. (2014). Cell-Autonomous Heterogeneity of Nutrient Uptake in White Adipose Tissue of Rhesus Macaques. Endocrinology.

[22] Chu M., Sampath H., Cahana D.Y., Kahl C.A., Somwar R., Cornea A., Roberts C.T., Varlamov O. (2014). Spatiotemporal dynamics of triglyceride storage in unilocular adipocytes. Mol. Boil. Cell.

[23] Kim D., Pertea G., Trapnell C., Pimentel H., Kelley R., Salzberg S.L. (2013). TopHat2: Accurate alignment of transcriptomes in the presence of insertions, deletions and gene fusions. Genome Boil..

[24] Liao Y., Smyth G.K., Shi W. (2014). featureCounts: An efficient general purpose program for assigning sequence reads to genomic features. Bioinformatics.

[25] Robinson M.D., McCarthy D.J., Smyth G.K. (2010). edgeR: A Bioconductor package for differential expression analysis of digital gene expression data. Bioinformatics.

[26] Kolahi K., Louey S., Varlamov O., Thornburg K. (2016). Real-Time Tracking of BODIPY-C12 Long-Chain Fatty Acid in Human Term Placenta Reveals Unique Lipid Dynamics in Cytotrophoblast Cells. PLoS ONE.

[27] Varlamov O., Somwar R., Cornea A., Kievit P., Grove K.L., Roberts J.C.T. (2010). Single-cell analysis of insulin-regulated fatty acid uptake in adipocytes. Am. J. Physiol. Metab..

[28] Somwar R., Roberts J.C.T., Varlamov O. (2011). Live-cell imaging demonstrates rapid cargo exchange between lipid droplets in adipocytes. FEBS Lett..

[29] Herman M., Peroni O.D., Villoria J., Schön M.P., Abumrad N.A., Blüher M., Klein S., Kahn B.B. (2012). A novel ChREBP isoform in adipose tissue regulates systemic glucose metabolism. Nature.

[30] Eissing L., Scherer T., Tödter K., Knippschild U., Greve J.W., Buurman W.A., Pinnschmidt H.O., Rensen S.S., Wolf A.M., Bartelt A. (2013). De novo lipogenesis in human fat and liver is linked to ChREBP-β and metabolic health. Nat. Commun..

[31] Varlamov O., Bethea C.L., Roberts C.T. (2014). Sex-specific differences in lipid and glucose metabolism. Front. Endocrinol. (Lausanne).

[32] Catalan V., Gómez-Ambrosi J., Rotellar F., Silva C., Gil M.J., Rodríguez A., Cienfuegos J.A., Salvador J., Frühbeck G. (2007). The obestatin receptor (GPR39) is expressed in human adipose tissue and is down-regulated in obesity-associated type 2 diabetes mellitus. Clin. Endocrinol..

[33] Petersen P.S., Jin C., Madsen A.N., Rasmussen M., Kuhre R.E., Egerod K.L., Nielsen L.B., Schwartz T.W., Holst B. (2011). Deficiency of the GPR39 receptor is associated with obesity and altered adipocyte metabolism. FASEB J..

[34] Reilly S., Saltiel A.R. (2017). Adapting to obesity with adipose tissue inflammation. Nat. Rev. Endocrinol..

[35] Olefsky J.M., Glass C.K. (2010). Macrophages, Inflammation, and Insulin Resistance. Annu. Rev. Physiol..

[36] Hotamisligil G.S. (2017). Inflammation, metaflammation and immunometabolic disorders. Nature.

[37] Newman J.C., Verdin E. (2014). Ketone bodies as signaling metabolites. Trends Endocrinol. Metab..

[38] Youm Y.-H., Nguyen K.Y., Grant R.W., Goldberg E.L., Bodogai M., Kim D., D’Agostino D., Planavsky N., Lupfer C., Kanneganti T.-D. (2015). The ketone metabolite β-hydroxybutyrate blocks NLRP3 inflammasome–mediated inflammatory disease. Nat. Med..

[39] Liu L., Zeng M., Stamler J.S. (1999). Hemoglobin induction in mouse macrophages. Proc. Natl. Acad. Sci. USA.

[40] Johannsen D.L., Knuth N.D., Huizenga R., Rood J.C., Ravussin E., Hall K.D. (2012). Metabolic slowing with massive weight loss despite preservation of fat-free mass. J. Clin. Endocrinol. Metab..

[41] Fothergill E., Guo J., Howard L., Kerns J.C., Knuth N.D., Brychta R., Chen K.Y., Skarulis M.C., Walter M., Walter P.J. (2016). Persistent metabolic adaptation 6 years after “The Biggest Loser” competition. Obesity.

[42] Greenway F.L. (2015). Physiological adaptations to weight loss and factors favouring weight regain. Int. J. Obes..

[43] Rossmeislova L., Malisova L., Kracmerova J., Stich V. (2013). Adaptation of human adipose tissue to hypocaloric diet. Int. J. Obes. (Lond).

[44] Reynisdottir S., Ellerfeldt K., Wahrenberg H., Lithell H., Arner P. (1994). Multiple lipolysis defects in the insulin resistance (metabolic) syndrome. J. Clin. Investig..

[45] Stich V., Harant I., De Glisezinski I., Crampes F., Berlan M., Kunesova M., Hainer V., Dauzats M., Rivière D., Garrigues M. (1997). Adipose Tissue Lipolysis and Hormone-Sensitive Lipase Expression during Very-Low-Calorie Diet in Obese Female Identical Twins1. J. Clin. Endocrinol. Metab..

[46] Sengenes C., Stich V., Berlan M., Hejnova J., Lafontan M., Pariskova Z., Galitzky J. (2002). Increased lipolysis in adipose tissue and lipid mobilization to natriuretic peptides during low-calorie diet in obese women. Int. J. Obes..

[47] Flechtner-Mors M., Ditschuneit H.H., Yip I., Adler G. (1999). Sympathetic modulation of lipolysis in subcutaneous adipose tissue: Effects of gender and energy restriction. J. Lab. Clin. Med..

[48] Hellström L., Reynisdottir S., Langin D., Rössner S., Arner P. (1996). Regulation of lipolysis in fat cells of obese women during long-term hypocaloric diet. Int. J. Obes. Relat. Metab. Disord. : J. Int. Assoc. Study Obes..

[49] Presta E., Leibel R.L., Hirsch J. (1990). Regional changes in adrenergic receptor status during hypocaloric intake do not predict changes in adipocyte size or body shape. Metabolism.

[50] Berlan M., Dang-Tran L., Lafontan M., Denard Y. (1981). Influence of hypocaloric diet on alpha-adrenergic responsiveness of obese human subcutaneous adipocytes. Int. J. Obes..

[51] Rozen R., Banegas E., Davilla M., Apfelbaum M. (1984). Effects of a very-low-calorie diet on adrenergic responsiveness in human adipose tissue. Int. J. Obes..

[52] Kather H., Wieland E., Fischer B., Wirth A., Schlierf G. (1985). Adrenergic regulation of lipolysis in abdominal adipocytes of obese subjects during caloric restriction: Reversal of catecholamine action caused by relief of endogenous inhibition. Eur. J. Clin. Investig..

[53] Kolehmainen M., Vidal H., Ohisalo J.J., Pirinen E., Alhava E., Uusitupa M.I.J. (2002). Hormone sensitive lipase expression and adipose tissue metabolism show gender difference in obese subjects after weight loss. Int. J. Obes..

[54] Löfgren P., Hoffstedt J., Näslund E., Wirén M., Arner P. (2005). Prospective and controlled studies of the actions of insulin and catecholamine in fat cells of obese women following weight reduction. Diabetologia.

[55] Tang Y., Wallace M., Gurmaches J.S., Hsiao W.-Y., Li H., Lee P.L., Vernia S., Metallo C.M., Guertin D.A. (2016). Adipose tissue mTORC2 regulates ChREBP-driven de novo lipogenesis and hepatic glucose metabolism. Nat. Commun..

[56] Minehira K., Vega N., Vidal H., Acheson K., Tappy L. (2004). Effect of carbohydrate overfeeding on whole body macronutrient metabolism and expression of lipogenic enzymes in adipose tissue of lean and overweight humans. Int. J. Obes..

[57] Strawford A., Antelo F., Christiansen M., Hellerstein M.K. (2004). Adipose tissue triglyceride turnover, de novo lipogenesis, and cell proliferation in humans measured with 2H2O. Am. J. Physiol. Metab..

[58] Solinas G., Borén J., Dulloo A. (2015). De novo lipogenesis in metabolic homeostasis: More friend than foe?. Mol. Metab..

[59] Yilmaz M., Claiborn K.C., Hotamisligil G.S. (2016). De Novo Lipogenesis Products and Endogenous Lipokines. Diabetes.

[60] Yore M.M., Syed I., Moraes-Vieira P.M., Zhang T., Herman M., Homan E.A., Patel R.T., Lee J., Chen S., Peroni O.D. (2014). Discovery of a class of endogenous mammalian lipids with anti-diabetic and anti-inflammatory effects. Cell.

[61] Moraes-Vieira P.M., Saghatelian A., Kahn B.B. (2016). GLUT4 Expression in Adipocytes Regulates De Novo Lipogenesis and Levels of a Novel Class of Lipids with Antidiabetic and Anti-inflammatory Effects. Diabetes.

[62] Pietiläinen K.H., Rog T., Seppänen-Laakso T., Virtue S., Gopalacharyulu P., Tang J., Rodríguez-Cuenca S., Maciejewski A., Naukkarinen J., Ruskeepää A.-L. (2011). Association of lipidome remodeling in the adipocyte membrane with acquired obesity in humans. PLoS Boil..

[63] Würtz P., Wang Q., Kangas A., Richmond R., Skarp J., Tiainen M., Tynkkynen T., Soininen P., Havulinna A.S., Kaakinen M. (2014). Metabolic Signatures of Adiposity in Young Adults: Mendelian Randomization Analysis and Effects of Weight Change. PLoS Med..

[64] Carbone L., Davis B.A., Fei S.S., White A., Nevonen K.A., Takahashi D., Vinson A., True C., Roberts C.T., Varlamov O. (2019). Synergistic Effects of Hyperandrogenemia and Obesogenic Western-style Diet on Transcription and DNA Methylation in Visceral Adipose Tissue of Nonhuman Primates. Sci. Rep..

[65] True C., Abbott D.H., Roberts J.C.T., Varlamov O. (2017). Sex Differences in Androgen Regulation of Metabolism in Nonhuman Primates. Adv. Exp. Med. Biol..

[66] Varlamov O., Chu M.P., McGee W.K., Cameron J.L., O’Rourke R.W., Meyer K.A., Bishop C., Stouffer R.L., Roberts J.C.T. (2013). Ovarian cycle-specific regulation of adipose tissue lipid storage by testosterone in female nonhuman primates. Endocrinology.

[67] Viguerie N., Montastier E., Maoret J.-J., Roussel B., Combes M., Valle C., Villa-Vialaneix N., Iacovoni J.S., Martínez J.A., Holst C. (2012). Determinants of Human Adipose Tissue Gene Expression: Impact of Diet, Sex, Metabolic Status, and Cis Genetic Regulation. PLoS Genet..

[68] Capel F., Klimčáková E., Viguerie N., Roussel B., Vítková M., Kováčiková M., Polak J., Kováčová Z., Galitzky J., Maoret J.-J. (2009). Macrophages and Adipocytes in Human Obesity: Adipose Tissue Gene Expression and Insulin Sensitivity During Calorie Restriction and Weight Stabilization. Diabetes.

[69] Giles E.D., Steig A.J., Jackman M.R., Higgins J.A., Johnson G.C., Lindstrom R.C., MacLean P. (2016). Exercise Decreases Lipogenic Gene Expression in Adipose Tissue and Alters Adipocyte Cellularity during Weight Regain After Weight Loss. Front. Physiol..

[70] Wajchenberg B.L. (2000). Subcutaneous and visceral adipose tissue: Their relation to the metabolic syndrome. Endocr. Rev..

